# Prefrontal tDCS modulates risk-taking in male violent offenders

**DOI:** 10.1038/s41598-024-60795-z

**Published:** 2024-05-02

**Authors:** Leandra Kuhn, Olivia Choy, Lara Keller, Ute Habel, Lisa Wagels

**Affiliations:** 1 Department of Psychiatry, Psychotherapy and Psychosomatics, Faculty of Medicine, RWTH Aachen, Pauwelsstr. 30, 52074 Aachen, Germany; 2 Department of Psychology, Nanyang Technological University, Singapore, Singapore; 3 Department of Child and Adolescent Psychiatry, Psychosomatics and Psychotherapy, University Hospital RWTH, Aachen, Germany; 4 Institute of Neuroscience and Medicine: JARA-Institute Brain Structure Function Relationship (INM 10), Research Center Jülich, Jülich, Germany

**Keywords:** tDCS, fMRI, Prefrontal cortex, Violent offenders, Risk-taking, Cognitive neuroscience, Human behaviour

## Abstract

Detrimental decision-making is a major problem among violent offenders. Non-invasive brain stimulation offers a promising method to directly influence decision-making and has already been shown to modulate risk-taking in non-violent controls. We hypothesize that anodal transcranial direct current stimulation (tDCS) over the right dorsolateral prefrontal cortex beneficially modulates the neural and behavioral correlates of risk-taking in a sample of violent offenders. We expect offenders to show more risky decision-making than non-violent controls and that prefrontal tDCS will induce stronger changes in the offender group. In the current study, 22 male violent offenders and 24 male non-violent controls took part in a randomized double-blind sham-controlled cross-over study applying tDCS over the right dorsolateral prefrontal cortex. Subsequently, participants performed the Balloon Analogue Risk Task (BART) during functional magnetic resonance imaging (fMRI). Violent offenders showed significantly less optimal decision-making compared to non-violent controls. Active tDCS increased prefrontal activity and improved decision-making only in violent offenders but not in the control group. Also, in offenders only, prefrontal tDCS influenced functional connectivity between the stimulated area and other brain regions such as the thalamus. These results suggest baseline dependent effects of tDCS and pave the way for treatment options of disadvantageous decision-making behavior in this population.

## Introduction

Violent crimes pose a significant burden on society as they involve enormous economic and societal costs. Existing evidence indicates that what leads someone to commit violent crimes comprises interactions of biological, psychosocial, and environmental factors^1^. Despite advances in our understanding of the etiology of violent offending, their link to effective treatment options is often missing. Popular therapy programs such as cognitive behavioral therapy may fail in certain groups more often, such as in violent offenders who tend to make risky decisions^2^. The exploration of biological pathways may advance our knowledge of the mechanisms of the different cognitive and emotional dysfunctions in violent populations, in order to shed light on ways to reduce these deficits.

One prominent explanatory biological pathway is described in the prefrontal dysfunction hypothesis^3^. Based on this hypothesis, studies have shown a hypoactivity of the prefrontal cortex in violent offenders which can be linked to emotion regulation as well as cognitive deficits, such as in the context of risky decision-making^4^. A promising non-invasive method that can alter neural functioning and thereby influence cognition and behavior is transcranial direct current stimulation (tDCS). Prefrontal tDCS has already been proven to be effective for neural modulation in various populations including criminal offenders^5,6^. In the current study, we aim to directly compare the effects of prefrontal tDCS on risky decision-making in violent offenders and non-violent controls at both the behavioral and neural level for the first time.

When decisions are made under risk, economic models state that individuals need to integrate information about possible wins and losses and their respective probabilities^7^. Individuals choose the option to which they assign the highest subjective value. Such situations are experimentally modeled by gambling tasks such as the Balloon Analogue Risk Task (BART)^8^. In the BART, participants inflate a virtual balloon to maximize their reward while facing the risk of explosion and losing the reward. High numbers of inflations have been linked to substance use or sexual risk behavior ^8^ and were associated with impulsive and psychopathic personality traits in a non-forensic sample^9^.

In offender samples, aberrant decision-making behavior may originate from general deficits in executive functioning^10–12^, cognitive control^13,14^, and emotion regulation^15,16^. A meta-analysis showed an increased propensity of aggressive individuals to engage in risky choices^2^. This propensity has been linked to different measures of aggression as well as poorer treatment outcomes^17^.

Only a few studies have examined the neural underpinnings of behavioral differences between offenders and non-offenders. Among other brain regions, the dorso-lateral prefrontal cortex (dlPFC), a central region for cognitive control and decision-making, showed aberrant activation patterns. Prehn et al. showed a diminished response in the anterior cingulate as well as the prefrontal cortex especially in emotionally hypo-reactive offenders during decision-making under risk^4^. Adolescents with conduct and substance use problems also showed hypoactivation in the prefrontal cortex, anterior insula, anterior cingulate and subcortical regions during risky decision-making^18^. This largely overlaps with the findings from a meta-analysis of individuals on the anti-social spectrum^19^.

As a non-invasive method, tDCS can alter cortical excitability through the subthreshold modulation of neuronal membrane potentials^20,21^. Anodal stimulation depolarizes the neuronal membrane and thereby increases spontaneous neuronal activity and the probability to induce an action potential^22,23^. Beneficial effects of unilateral or bilateral stimulation of the dlPFC have been found in healthy participants^24^ as well as patient groups with ADHD or addiction disorders^25–28^. However, across studies, unilateral stimulation was identified to reduce risk-taking behavior more effectively than bilateral protocols^29^, with right anodal/left cathodal stimulation as the most effective protocol^30^. Using either right anodal/left cathodal or left anodal/right cathodal dlPFC stimulation, a reduction of risk-taking in the BART was reported in a healthy sample^31^. Anodal stimulation of the right dlPFC also lowered risk-aversion in a gain frame condition, leading to optimized decision-making behavior^32^. Interestingly, in older adults as well as marijuana users, anodal tDCS over the dlPFC increased risk-taking^33,34^.

In the BART, earnings are largest if the participant finds the optimal number of button presses. Therefore, general task performance such as the total earnings can indicate successful decision-making. While tDCS seems to influence earnings in the BART as well^35^, it is unknown if tDCS can optimize decision-making in criminal offenders. Some support for this notion comes from the finding that in paedophilic offenders, cognitive control was improved after the stimulation of the left dlPFC compared to sham stimulation^36^. Offenders’ moral decision-making as well as self-reported aggression were also influenced advantageously by prefrontal tDCS^6,37,38^. In line with these findings, recent literature reviews on tDCS effects on aggression in healthy as well as criminal subjects found reductions in aggressive behavior using both self-report and behavioral measures^39,40^. In participants with psychopathic traits, increased inhibitory control due to tDCS was positively correlated with greater psychopathy scores^41^. In violent offenders, dlPFC stimulation reduced brain activity during emotion regulation^5^. Although investigation of brain activity after tDCS over the dlPFC in non-violent populations indicates increased activation during risk-taking in the BART at the stimulation site as well as the anterior cingulate^42^, tDCS may affect the whole brain via connectivity changes^42,43^ in both non-violent and offender groups.

In the current study, we compare the effects of prefrontal tDCS on risky decision-making in violent offenders and non-violent controls at both the behavioral and neural level for the first time. The goal of this study is to demonstrate that tDCS can be used to beneficially modulate the neural and behavioral correlates of risk-taking in a sample of violent offenders. Specifically, this study tests whether anodal stimulation of the right dlPFC facilitates its cortical excitability, reflected by increased right dlPFC activation during risk-taking after the active compared to the sham tDCS condition. We anticipate a tDCS-induced change in the connectivity of the dlPFC and other regions related to risk-taking. Thus, we expect to improve cognitive control and reduce risk-taking after active tDCS. We hypothesize that offenders show more risky decision-making than non-violent controls and hence, that prefrontal tDCS will induce stronger changes in the neural and behavioral correlates of risk-taking in the offender group.

## Methods

### Participants

We recruited 22 male violent offenders in the age range from 23 to 52 years (*M* = 37.2, *SD* = 8.6) at the parole office in Aachen, Germany. Inclusion criteria for offenders included commission of at least two violent crimes. The distribution of index crimes was as follows: 5 offenders committed or attempted murder or manslaughter, 11 committed assault or physical injury, 5 committed (armed) robbery, and one committed sexual assault. Due to the high rate of psychiatric diagnoses in this group, only participants reporting acute alcohol and drug abuse were excluded. For further information on the lifetime psychiatric diagnoses and medication of the violent offender group, please see supplementary Table 1. The non-violent control group included 24 men aged between 19 and 58 years (*M* = 31.4, *SD* = 10.5) without any former or current neurological or psychiatric disease. Non-violent controls were recruited by advertisements in Aachen and did not have any criminal record. For both groups, exclusion criteria for MRI were applied, such as metal implants and large-scale tattoos. Participants with neurological diseases were excluded. All participants were right-handed.

### Ethics

Participation was voluntary and participants gave written and informed consent. The experimental procedures were in accordance with the declaration of Helsinki (World Medical Association, 2013) and the study was approved by the Ethics Committee of the Medical Faculty of the RWTH Aachen University. The trial protocol was registered at ClinicalTrials.gov (NCT03036683) on 01/02/2017.

### Study design

We implemented a double-blind sham-controlled cross-over design. Measurements took place in the Department of Psychiatry, Psychotherapy and Psychosomatics of the University Hospital RWTH Aachen, Germany. Participants visited the laboratory twice and underwent both active and sham tDCS in a randomized order with at least 48 h in between. This time period has been shown to be sufficient to wash out tDCS effects and prevent carry over effects^44–46^. The cross-over design increases statistical power and is advantageous for heterogeneous groups since every subject serves as a control for himself. The average time interval between measurements was five days with a maximum of two weeks.

### Procedure

Non-violent controls were interviewed about demographic information and psychiatric symptoms using the SCID light^47^. They filled in questionnaires, performed a verbal intelligence test, and tests of executive functioning (Trail Making Test-A/-B, digit span forward and backwards)^48^. We assessed demographics, criminal records and psychopathology in the offender group at a separate appointment, which included a structured interview on their committed crimes, the German versions of DIPS (Diagnostic Interview for Mental Disorder)^49^, the PCL-R (Hare Psychopathy Checklist—Revised; German version)^50^, an assessment of verbal intelligence (WST)^51^, and various self-report questionnaires. The self-report questionnaires included German versions of the Becks Depression Inventory (BDI-II)^52^, the Aggression Questionnaire^53^, the Proactive Reactive Aggression Questionnaire^54^, the Barratt Impulsiveness Scale (BIS-11)^55^, and the Psychopathic Personality Inventory-Revised (PPI-R)^56^.

Prior to the stimulation, participants were told that they could increase their financial compensation with their performance in the fMRI tasks by earning the money saved in the total account during the BART. In fact, all individuals received the same fixed amount of additional money after debriefing.

**Figure 1:**
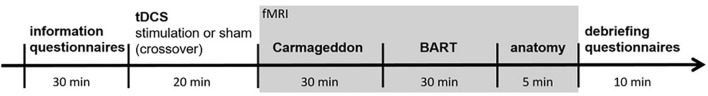
Procedure of the experiment.

Participants in both groups practiced the BART and then performed an alternating 1-back, 2-back or 3-back working memory task^57^ during the 20 min of stimulation. The working memory task was only applied to facilitate stimulation effects in the dlPFC and the results were not of interest for the current study. It has been shown that tasks that activate the stimulated brain region can enhance the stimulation effect compared to being at rest during the stimulation^58^. After that, participants went to the 3 Tesla scanner to perform a computer car race game (Carmageddon, Torus Games, Bayswater, Australia, 2000, reported elsewhere) followed by the BART and an anatomical scan (Fig. 1). In total, approximately 40 min passed in between the tDCS termination and the BART. After the session, participants completed a tDCS questionnaire assessing the blinding of participants and possible adverse effects as well as affective responses using the Positive and Negative Affect Schedule (PANAS)^59^. The results of the PANAS are reported in the supplementary material (supp. Tables 2 and 3).

### Stimulation protocol

A DC-stimulator (neuroCare Group GmbH, Munich, Germany) was used to stimulate the right dlPFC. A code system assigned the participant to the sham and active condition in a randomized order, keeping both the participant and experimenter blinded. The electrodes were positioned according to the international 10–20 EEG system. The anode (5 × 5 cm) was placed at the F4 position to target the right dlPFC. The cathode (10 × 10 cm) served as a reference electrode. It was placed over the left eyebrow with at least 7 cm distance to the anode to avoid a current flow over the scalp. The electrodes were enveloped in sponges soaked in 0.9% NaCl to increase conductivity and were attached using rubber bands. After a ramp-up phase of 40 s, a direct current flow of 2 milliampere lasted for 20 min. Participants reported only mild sensations of tickling or itching during the stimulation and could not differentiate between active and sham stimulation (χ^2^(1) = 1.51, *p* = 0.219).

### BART

The applied BART is a well-established computerized risk-taking task adapted for the use in the MRI^60,61^. Participants had to inflate virtual balloons to earn an increasing amount of money. In case of an explosion, the money from that trial was lost and a new balloon appeared. Control balloons would not explode or fetch money. Before each inflation, participants had the option to save their temporal reward to their permanent account. For further details and visualization, see supplementary Fig. 1. The BART lasted approximately 30 min.

### Behavioral analysis

Behavioral analyses of different BART parameters were conducted using R Studio and the lme4 package^62^. The primary outcome was measured by the mean number of pumps in successfully cashed out trials (*adjusted pumps*). Explosion trials were excluded for the calculation since they represent the random structure of the program, but the number of explosion trials was used as covariate to control for their indirect influence. The secondary outcome was the total earnings. Mixed models for both dependent variables were computed including group (controls vs. offenders), condition (sham vs. active stimulation) and session (first vs. second measurement) as fixed factors, as well as a random intercept and the covariate of explosions for the adjusted pump model. All main effects and interactions were modeled based on theoretical assumptions (model space) and selected based on goodness of fit tests comparing the models with the anova function of the lme4 package. For an overview on the model space, see Supplementary Tables 4, 5. For both dependent variables, a model with the two-way interactions of group and stimulation as well as stimulation and session were selected. Type III analysis with Satterthwaite methods were performed for the selected models. Tukey correction at an original α = 0.05 was applied for post-hoc pairwise comparisons and degrees of freedom were adjusted via the Kenward-Roger method.

Bonferroni corrected Pearson correlations of the adjusted pumps and different personality scores were computed for each subgroup. Additionally, sample characteristics were compared between groups using independent sample t-tests.

### Image acquisition and analysis

Data acquisition, preprocessing and first level modelling are described in the supplements.

For the whole brain analysis, a GLM was fitted using a flexible factorial design on the parametric modulation of the BOLD response modeled via the balloon size (representing increasing risk). Group (offenders vs. controls), tDCS condition (active vs. sham), as well as session (first vs. second) were included as fixed factors. Additionally, a random intercept was included in the model. A region of interest (ROI) analysis of the right dlPFC as the target area of stimulation was conducted using the same design. An inclusive mask of Brodmann areas 9 and 46 was created using the WFU PickAtlas^63^. Additionally, the activation in this mask was extracted and included in the correlational analysis.

To examine changes in functional task-dependent connectivity related to risky decision-making after tDCS stimulation, we conducted a psychophysiological interaction (PPI) analysis using the generalized PPI toolbox for SPM^64^. Therefore, we defined the rdlPFC as the seed region, again using the aforementioned mask. For each individual, the deconvolved time series of this seed region was extracted. The event types (inflation vs. baseline) were convolved with the canonical hemodynamic response function (HRF) to create the psychological regressor. An interaction term (reflecting the PPI) was calculated by multiplying the time series of the psychological variable with the time series of the seed. For the group-level analysis, we applied the same flexible factorial design reported for brain activation including the factors group, session, and condition. Whole brain results were family-wise error (FWE) corrected at cluster level using a height threshold of *p* < 0.05 and a cluster-defining threshold of *p* < 0.001.

## Results

### Questionnaires

Offenders reported elevated levels of depression, aggression, impulsivity as well as psychopathy compared with controls (Table 1).

**Table 1:** Sample characteristics.

	Offenders	Controls	*t*	*p*
Age	37.18 (8.61)	31.38 (10.49)	2.89	0.005
Verbal IQ (WST)	97.36 (10.10)	107.33 (11.21)	− 4.47	< 0.001
Depression (BDI-II)	10.41 (8.79)	1.83 (3.71)	6.08	< 0.001
Aggression (AQ)	76.14 (15.76)	48.18 (8.38)	10.33	< 0.001
Reactive aggression (RPQ)	12.00 (5.28)	4.00 (2.25)	9.42	< 0.001
Proactive aggression (RPQ)	8.36 (6.27)	0.52 (1.03)	8.36	< 0.001
Impulsivity (BIS11)	63.95 (10.13)	55.33 (5.08)	5.22	< 0.001
Attentional	15.23 (3.34)	12.29 (1.76)	5.33	< 0.001
Motor	22.63 (3.97)	20.08 (3.15)	3.43	0.001
Nonplanning	26.09 (4.95)	22.96 (3.47)	3.54	0.001
PPI-R	309.14 (36.00)	268.22 (24.21)	6.43	< 0.001
PPI-I: Fearless dominance	104.67 (15.73)	110.88 (14.81)	− 1.93	0.057
Social potency	44.45 (9.15)	45.63 (8.26)	− 0.65	0.521
Stress immunity	40.71 (6.77)	47.75 (6.46)	− 5.04	< 0.001
Fearlessness	19.28 (5.06)	17.50 (4.88)	1.66	0.099
PPI-II: Self-centered impulsivity	167.48 (21.70)	128.68 (18.10)	9.02	< 0.001
Carefree nonplanfulness	33.33 (7.59)	28.95 (4.07)	3.46	0.001
Impulsive nonconformity	64.63 (14.78)	48.42 (12.70)	5.47	< 0.001
Blame externalization	37.10 (6.47)	22.04 (5.81)	11.50	< 0.001
Machiavellian egocentricity	37.18 (6.47)	31.00 (4.61)	5.24	< 0.001
Coldheartedness	32.57 (7.61)	34.58 (5.94)	− 1.41	0.163

Mean values and standard deviations for offenders and controls.

Because verbal IQ and BDI-II scores were potentially confounding variables and differed between groups, correlations with the adjusted pumps and total earnings in the BART were computed separately for each group. No significant correlation emerged in either group (Supplementary Table 6).

### BART

Goodness of fit tests (Table 2) showed that for both adjusted pumps and earnings, the models which included the main effects of group, stimulation, and session as well as the interaction of group and stimulation, and stimulation and session were significantly better than the other models of the given model space (see Supplementary Tables 4 and 5 for the full model space).

**Table 2:** Models for adjusted pumps and earnings with goodness of fit test statistic.

		npar	AIC	BIC	logLikelihood	deviance	Chisq	df	p
ad. Pumps	Model1	8	183.95	203.76	− 83.973	167.95			
	Model2	8	189.96	203.78	− 86.98	173.96	< .001	0	
	Model3*	**9**	**182.03**	**204.33**	** − 82.015**	**164.03**	**9.9302**	**1**	**0.00163**
	Model4	10	183.97	208.74	− 81.984	163.97	0.062	1	0.8034
	Model_Full	11	185.2	212.45	81.598	163.2	0.7712	1	0.3799
Earnings	Model1	7	552.5	569.92	− 269.25	538.5			
	Model2	7	557.79	575.21	− 271.89	543.79	< .001	0	
	Model3*	**8**	**553.88**	**573.79**	** − 268.94**	**537.88**	**5.905**	**1**	**0.0151**
	Model4	8	553.88	573.79	− 268.94	537.88	0.0037	0	0.9516
	Model_Full	10	557.66	582.55	− 268.83	537.66	0.2171	1	0.6413

*Statistically significant model of which effects are reported in the results.

Significant values are in bold.

**Figure 2:**
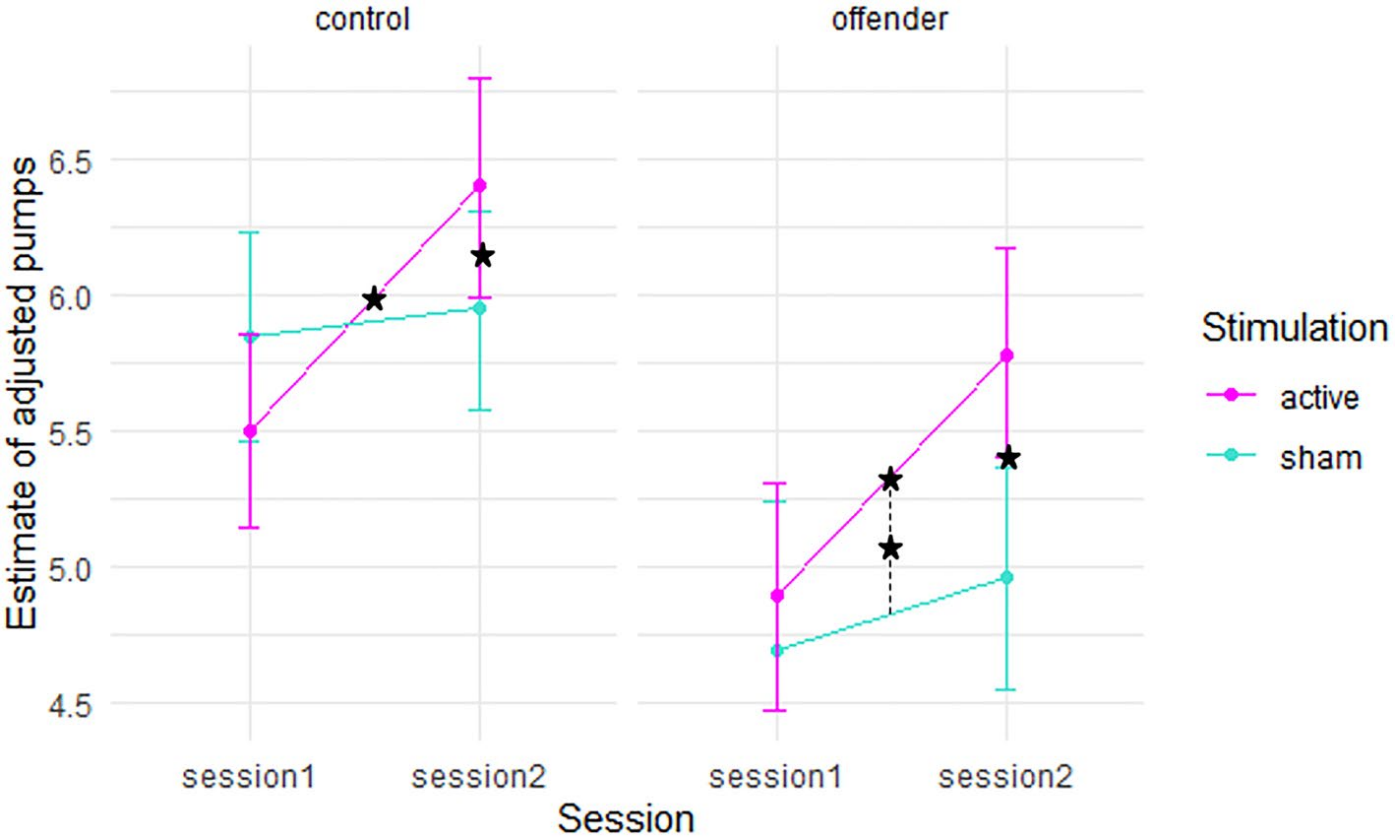
Estimated marginal means of adjusted pumps (mean number of balloons in non-explosion trails) and 95% confidence intervals for session one and two in the active and sham stimulation condition separated for the control and offender group. Asterisks indicate significant difference at p < 0.05 corrected for multiple comparisons applying the Tukey method.

Concerning adjusted pumps, the main effect of group was significant (*F*(1,42.446) = 15.541, *p* < 0.001) with controls having a higher number of pumps than offenders. In addition, the interaction of session and stimulation was significant (*F*(2,42.463) = 15.273, *p* =  < 0.001) and the interaction of group and stimulation (*F*(1,41.861) = 3.881, *p* = 0.055) was marginally significant (Fig. 2).

Comparisons of the estimated marginal means (Table 3) showed that there was a significant difference between the active and sham stimulation conditions in session two (*t*(60.6) = − 2.803 *p* = 0.0334), but not in session one (*t*(59.8) = 0.708, *p* = 0.894). Session one and two differed in the active condition (*t*(61.0) = − 3.881, *p* = 0.0014), but not during the sham condition (*t*(59.5) = − 0.0434, *p* = 0.996). The marginally significant interaction of group and stimulation showed a significant difference between controls and offenders in the sham condition (t(58.8) 4.46, *p* = 0.002) and to a smaller degree in the active condition (*t*(58.8) = 4.46, *p* = 0.045). The adjusted pumps in the control group did not differ between sham and active conditions (*t*(40.6) = − 0.396, *p* = 0.979), but in the offender group, the adjusted pumps differed after participants underwent active and sham stimulation (*t*(42.3) = -3.042, *p* = 0.020).

**Table 3:** Raw scores, estimated marginal means, standard error of mean (SEM), and 95% confidence intervals of adjusted pumps for the interaction of group and stimulation as well as session and stimulation condition.

Factor 1	Factor 2	Raw mean scores	Estimated marginal means	SEM	df	Lower CI	Upper CI
Sham	Control	5.90	5.90	0.15	57.4	5.6	6.2
	Offender	4.93	4.91	0.16	59.6	4.58	5.23
Active	Control	5.94	5.95	0.15	58.4	5.64	6.25
	Offender	5.37	5.34	0.16	60.1	5.01	5.67
Sham	Sess1	5.38	5.35	0.16	57.7	5.04	5.67
	Sess2	5.55	5.45	0.16	60.2	5.14	5.77
Active	Sess1	5.23	5.19	0.16	60.9	4.87	5.51
	Sess2	6.14	6.09	0.16	58.9	5.77	6.41

The adjusted pumps correlated negatively with the RPQ (*r* = − 0.416, *p* < 0.001) and the AQ (*r* = − 0.474, *p* < 0.001) sum scores. No significant correlations emerged with psychopathy scores (PPI-R, *r* = − 0.231, *p* = 0.046) or impulsivity (BIS-11, *r* = − 0.244, *p* = 0.038). In the subgroups, no significant correlations remained (supplementary Tables 7–9). Notably, in the offender group a large effect of (*r* = − 0.531, *p* = 0.013) with the AQ was observed (Cohen, 1988) at uncorrected level.

**Figure 3:**
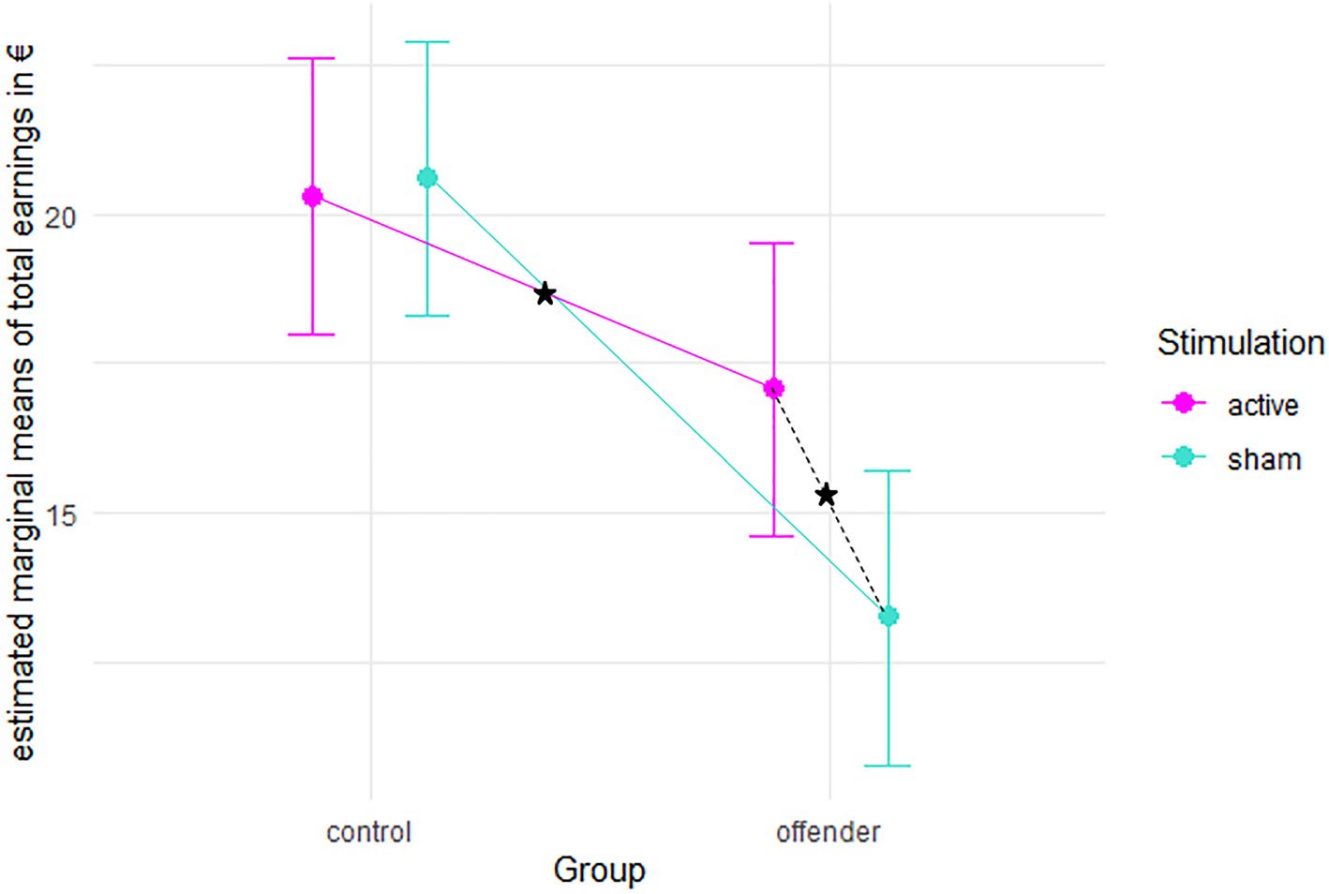
Estimated marginal means of total earnings (calculated as sum across all cash trials) and 95% confidence intervals for the active and sham stimulation condition separated for the control and offender group. Asterisks indicate significant difference at p < 0.05 corrected for multiple comparisons applying the Tukey method.

Total earnings were significantly influenced by group (*F*(1,42.59) = 12.85, *p* < 0.001), stimulation (*F*(1,40.93) = 4.64, *p* = 0.037), and session (*F*(1,40.81) = 15.92, *p* < 0.001). The interaction of group and stimulation (Fig. 3) was significant as well (*F*(1,41,029) = 6.28, *p* = 0.016), but the interaction of session and stimulation was not (*F*(1,42.49) = 0.58, *p* = 0.451).

Post hoc comparisons showed that the controls and offenders significantly differed in their total earnings in the sham condition (*t*(65.2) = 4.36, *p* < 0.001), but not in the active condition (*t*(66.1) = 1.94, *p* = 0.222). The sham and active condition did not differ in this outcome variable in controls (*t*(40.1) = 0.272, *p* = 0.993), but there were differences across conditions in the offender group (*t*(41.4) = -3.133, *p* = 0.016) (see Table 4 for estimated marginal means and confidence intervals). Total earnings correlated significantly with the adjusted pumps (*r* = 0.872, *p* < 0.001) and the RPQ (*r* = -0.359, *p* = 0.001) in the whole sample. In the subgroups, no significant correlations of the total earnings and personality measures were observed.

**Table 4:** Raw scores, estimated marginal means, standard error of mean (SEM), and 95% confidence intervals of the total earnings for the interaction of stimulation condition and group.

Factor 1	Factor 2	Raw mean scores	Estimated marginal means	SEM	df	Lower CI	Upper CI
Sham	Control	20.73	20.6	1.15	64.0	18.3	22.9
	Offender	13.28	13.3	1.23	66.1	10.8	15.7
Active	Control	20.17	20.3	1.15	64.0	18.0	22.6
	Offender	17.65	17.1	1.24	67.9	14.6	19.5

### Functional imaging

Inflating balloons in the BART compared to saving led to increased activation in the midcingulate cortex (MCC), insula, striatal, parietal as well as prefrontal regions. In addition, cerebellar and occipital regions were involved (supplementary Table 10).

**Figure 4:**
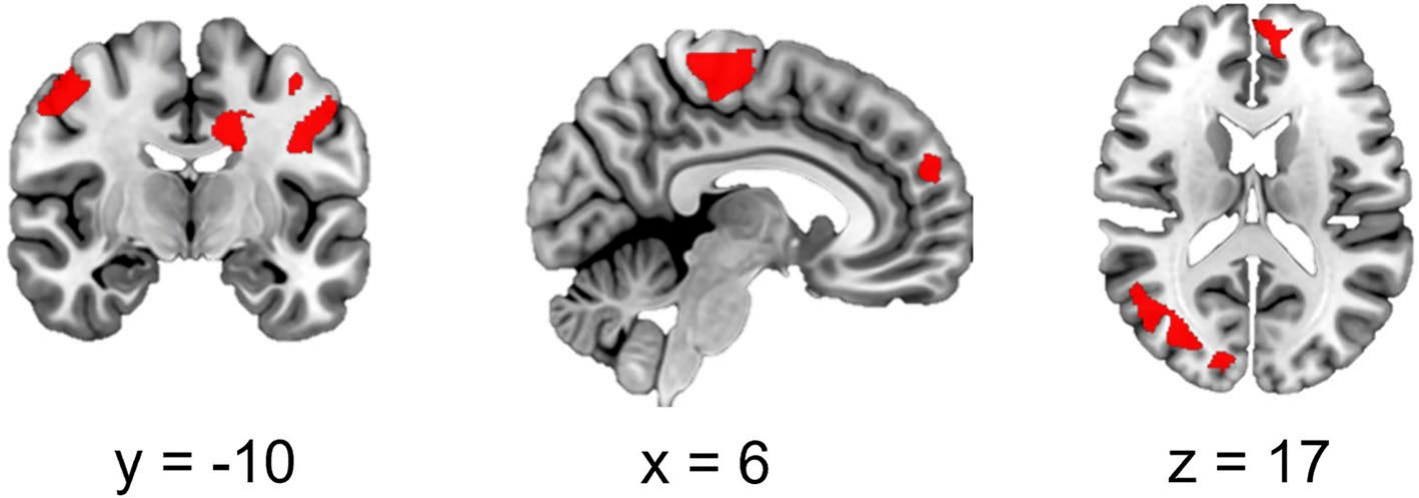
Main effect of group (offenders > controls) in risk-related neural activity (risky > control). Cluster-level FWE-corrected, *p* < 0.05 (at cluster defining threshold at p < 0.001), minimal cluster threshold *k* = 3018.

The group comparison yielded a more pronounced risk-related neural activation (risky > control balloons) in the offender compared to the control group (Fig. 4, Table 5). Differences were detected in the motor cortex, dorsolateral and medial frontal cortex, the cuneus, as well as in occipital areas. The opposite contrast did not indicate any significant cluster that was more activated in controls compared to offenders.

**Table 5:** MNI coordinates of peak voxels in significant clusters of the contrast offenders > controls.

Cluster	Max	Region	*x*	*y*	*z*	*T*	*k*
Cluster 1	1	R Paracentral Lobule	0	− 26	68	6.50	15,037
	2	R Precentral Gyrus	42	− 16	52	6.46	
	3	R Postcentral Gyrus	52	− 14	52	6.22	
	4	L Superior Parietal Lobule	− 22	− 38	62	6.05	
	5	L Postcentral Gyrus	− 20	− 30	70	5.98	
Cluster 2	1	L Superior Medial Gyrus	− 4	60	30	6.81	3944
	2	R Superior Frontal Gyrus	16	42	42	6.04	
	3	L Superior Frontal Gyrus	− 20	32	48	5.80	
	4	L Middle Frontal Gyrus	− 28	16	50	5.45	
	5	L Posterior-Medial Frontal	− 12	20	66	5.21	
Cluster 3	1	R Superior Occipital Gyrus	26	− 80	18	5.88	3018
	2	R Cuneus	14	− 92	16	5.83	
	3	R Middle Temporal Gyrus	48	− 72	8	5.58	
	4	R Middle Occipital Gyrus	46	− 74	6	5.56	
	5	L Superior Occipital Gyrus	− 14	− 88	18	5.23	

Anatomical labels were derived from to the JuBrain Anatomy toolbox for SPM (Eickhoff et al. 2005).

Cluster level FWE-corrected, *p* < 0.05, minimal cluster threshold *k* = 3018.

### tDCS effects

**Figure 5:**
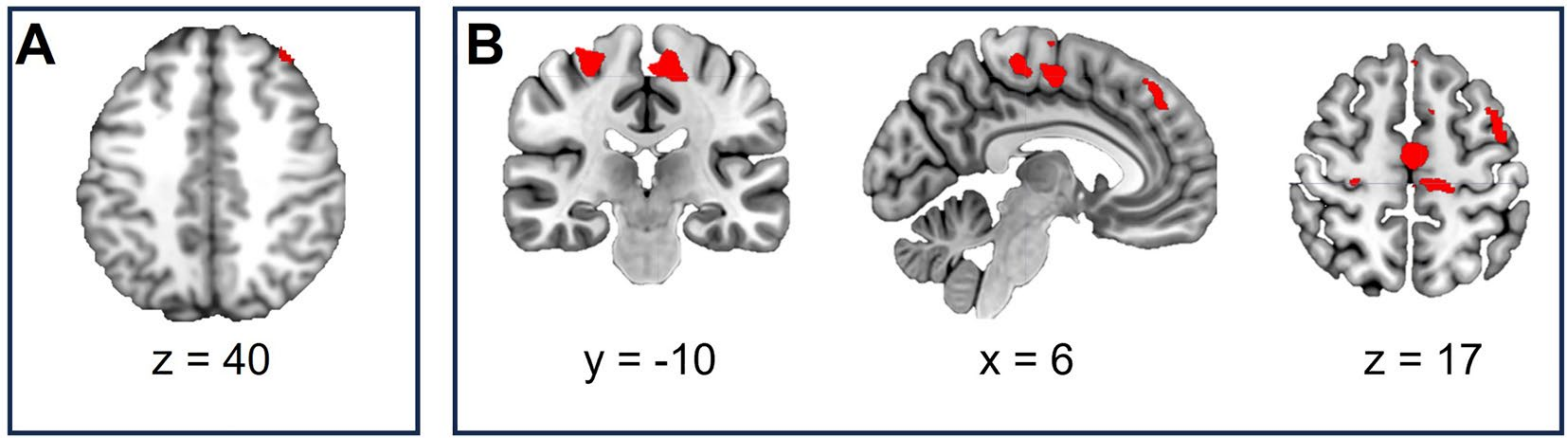
(**A**) ROI analysis of the right dlPFC, active > sham in the offender group, FWE-corrected, p < 0.05; (**B**) Risk-related neural activity increases after sham compared to active stimulation in the offender group. Cluster-level FWE-corrected, *p* < 0.05 (at cluster defining threshold at p < 0.001), minimal cluster threshold *k* = 225.

We performed a ROI analysis in the right dlPFC to test if active stimulation increased activity in the target area. There was no main effect of condition (active > sham), but a significant interaction of tDCS condition × group at a threshold of *p* < 0.001 uncorrected. The reported interaction holds significance only without adjustment for multiple comparisons, which has to be regarded with caution. Nevertheless, directed t-tests showed increased activation after active stimulation compared to sham in the offender group (*x* = 36 *y* = 42 *z* = 40, *t* = 4.46, *k* = 26) at a threshold of *p* < 0.001 uncorrected and applying FWE-correction at p < 0.05. In the control group, the stimulation condition had no significant effect on right dlPFC activity (t = 0.48, p_uncorr_ = 0.272, p_FWE_ = 1). Activity in this region correlated positively with the adjusted pumps in the whole sample (*r* = 0.289, *p* = 0.008) only at uncorrected level across both groups. On the whole-brain level, the contrast active > sham yielded no significant results across groups. Directed t-tests revealed significant activation patterns of the contrast sham > active in the offender but not in the control group. After sham compared to active stimulation, offenders showed greater activation in the MCC as well as motor regions (Fig. 5, Table 6).

**Table 6:** MNI coordinates of peak voxels in significant clusters of the contrast sham > active in offenders.

Cluster	Max	Region	*x*	*y*	*z*	*T*	*k*
Cluster 1	1	L Posterior-Medial Frontal	− 4	− 8	56	5.01	3030
	2	R Posterior-Medial Frontal	10	− 14	72	4.89	
	3	R Precentral Gyrus	28	− 24	58	4.89	
	4	L MCC	− 10	− 4	36	4.71	
	5	L Paracentral Lobule	− 2	− 32	60	4.69	
	6	R Paracentral Lobule	12	− 28	68	4.60	
	7	R Postcentral Gyrus	20	− 42	70	4.21	
Cluster 2	1	L Superior Medial Gyrus	− 2	40	46	5.20	498
	2	R Superior Medial Gyrus	8	40	50	4.43	
	3	L Posterior-Medial Frontal	− 6	24	50	3.90	
Cluster 3	1	L Precentral Gyrus	− 44	4	58	5.50	466
	2	L Postcentral Gyrus	− 54	− 8	42	4.07	
	3	L Middle Frontal Gyrus	− 30	14	54	3.89	
Cluster 4	1	L Angular Gyrus	− 50	− 58	40	4.20	264
	2	L Middle Occipital Gyrus	− 34	− 68	38	4.02	
	3	L Superior Parietal Lobule	− 26	− 76	52	3.42	
Cluster 5	1	L Posterior-Medial Frontal	− 14	12	64	5.01	225
	2	R Posterior-Medial Frontal	2	10	70	4.05	

Anatomical labels were derived from to the JuBrain Anatomy toolbox for SPM (Eickhoff et al. 2005).

Cluster-level FWE-corrected, p < 0.05, minimal cluster threshold k = 225.

### Psychophysiological Interactions

**Figure 6:**
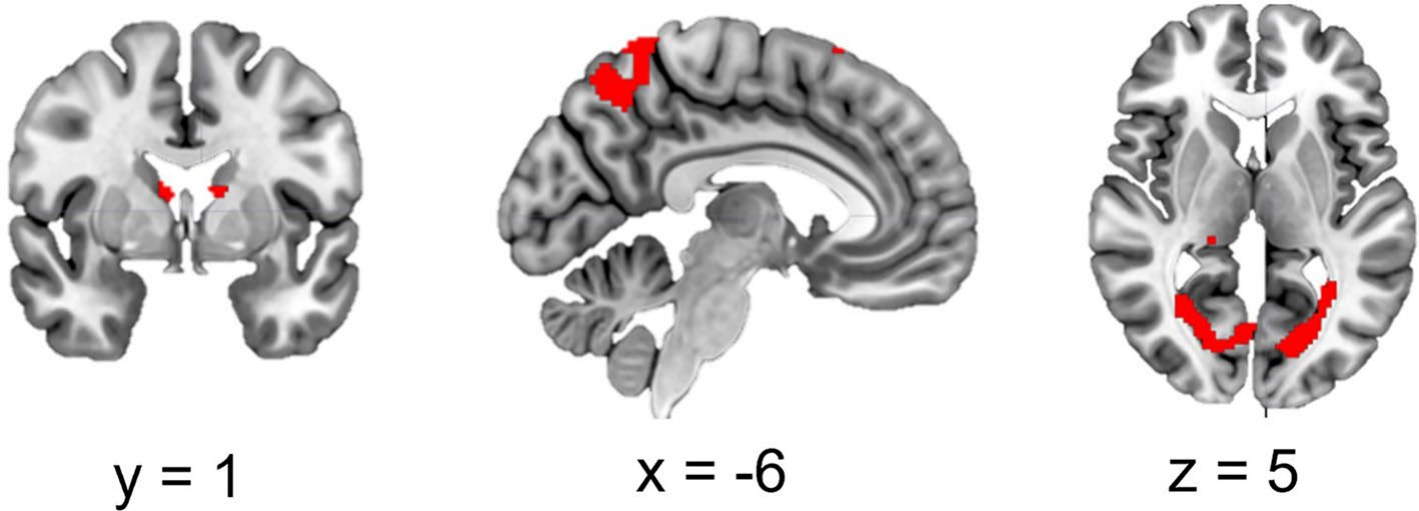
Clusters that reflect increased connectivity with the right dlPFC after active compared to sham stimulation in offenders. Cluster-level FWE-corrected, *p* < 0.05. (at cluster defining threshold at p < 0.001), minimal cluster threshold *k* = 227.

In the offender group, the PPI analysis revealed significant risk-related changes after active compared to sham stimulation in neural connectivity of the right dlPFC and other prefrontal and parietal regions, as well as the bilateral thalamus as shown in Fig. 6 and Table 7. No connectivity changes were observed for the control group.

**Table 7:** MNI coordinates of peak voxels in significant clusters in the PPI analysis of the contrast active > sham in offenders.

Cluster	Max	Region	*x*	*y*	*z*	*T*	*k*
Cluster 1	1	L Precuneus	− 4	− 52	72	6.47	1829
	2	R Precuneus	8	− 58	52	5.52	
	3	R Superior Parietal Lobule	22	− 72	60	5.50	
	4	R SupraMarginal Gyrus	54	− 46	42	5.33	
	5	R Inferior Parietal Lobule	30	− 50	50	5.31	
	6	R Angular Gyrus	38	− 66	54	5.16	
Cluster 2	1	R Calcarine Gyrus	20	− 72	6	6.44	865
	2	R Lingual Gyrus	28	− 56	2	5.43	
	3	R Thalamus	16	− 34	2	5.16	
	4	R Cuneus	14	− 90	14	3.96	
	5	L Calcarine Gyrus	0	− 80	14	3.89	
Cluster 3	1	L Middle Frontal Gyrus	− 24	26	62	6.20	829
	2	L Posterior-Medial Frontal	− 2	16	70	6.05	
Cluster 4	1	L Superior Parietal Lobule	− 36	− 46	68	5.71	462
	2	L Inferior Parietal Lobule	− 54	− 40	56	4.47	
	3	L Postcentral Gyrus	− 40	− 36	68	3.57	
Cluster 5		L Calcarine Gyrus	− 18	− 78	4	6.51	444
Cluster 6	1	L Superior Occipital Gyrus	− 20	− 94	20	5.67	346
	2	L Middle Occipital Gyrus	− 30	− 78	26	5.59	
Cluster 7	1	L Thalamus	− 14	− 2	6	6.16	236
	2	L Pallidum	− 22	− 6	6	3.68	
Cluster 8		R Middle Occipital Gyrus	36	− 82	24	5.88	227

Anatomical labels were derived from the JuBrain Anatomy toolbox for SPM (Eickhoff et al. 2005).

Cluster-level FWE-corrected, *p* < 0.05, minimal cluster threshold *k* = 227.

## Discussion

We tested the hypothesis that non-invasive brain stimulation may have the potential to reduce aggression in violent offenders. Indeed, tDCS of the right dlPFC affected decision-making and its neural underpinnings in violent offenders. Unexpectedly, offenders compared to non-violent controls overall behaved risk-averse in the BART, which led to poorer task outcomes. After active tDCS, this difference between offenders and non-violent controls was reduced as offenders showed riskier decision-making and thereby improved their performance after receiving active compared to sham stimulation. Active tDCS also only affected the offender and not the control group at the neural level underlining the behavioral modulation. In the control group, tDCS neither affected neural activity nor decision-making behavior.

Anodal prefrontal tDCS compared to sham stimulation decreased activation in different brain regions only in the offender and not in the control group. Reduced activity is in line with previous results on emotion regulation in offenders^5^. Since MCC activity changes have been related to risk-taking as well as threat processing^65,66^, we speculate that its deactivation contributes to the observed decrease of risk-avoidance in offenders. Our results complement findings that suggest complex tDCS effects including activation and deactivation depended on prior brain states^67,68^.

As in other studies^42,69^, seed-based connectivity of the right dlPFC showed extensive effects of tDCS only in the offender group specifically in subcortical regions such as the thalamus as well as occipital regions. This network re-organization may have supported observed performance improvements. The thalamus has also been linked to aggression in offender and patient samples^70,71^. It is an important hub for the organization of multiple cortical brain networks thereby being critical for integrating diverse information^72^.

Our behavioral results, showing a reduced rate of successful trials and probably more cautious approach behavior in violent offenders than in the control group, may query the image of offenders as impulsive and reckless individuals that are often linked to high aggression or clinical concepts of psychopathy^73^. This behavioral difference might not be influenced primarily by aggressiveness and impulsivity but cognitive processes that are important for optimal decision-making. In the violent offender group, IQ levels were lower than in the control group. Although we do not have an estimation for transfer or learning abilities, but only assessed verbal intelligence, this might point to generally lower cognitive abilities in the criminal offender group. Possibly, there were more problems in learning and estimating an optimal decision behavior in this group not due to reckless behavior, but due to other mental abilities that were missing for a good task performance. Although Snowden et al. found a correlation of specific psychopathic traits with risk-taking behavior in the BART, they did not find increased risk-taking in offenders compared to a community sample^74^. Others only reported minor differences comparing emotional hyperreactive offenders and controls^4^. In our study, psychopathy was weakly related to risk-taking only across the whole sample, which reflects the mixed^75^ and null findings^76^ of others. Still, direct comparisons of risk-taking in offenders and non-violent control groups remain scarce, and the present results contribute to filling this gap.

The reduced riskiness may question the ecological validity of the pumps in the BART in this population. While many studies found robust correlations between the BART performance and other risk indices in healthy controls^8,77^, no correlations with self-reported real life risk-taking were found in imprisoned inmates^76^. This is similarly supported in our findings because we did not find a correlation with self-reported impulsivity. We suggest that the number of pumps in the BART may reflect the ability to adapt a behavior to maximize rewards. Offenders had more difficulties adapting, as indicated by smaller earnings. Adapting choices in the BART requires impulse control, working memory, and learning capacities which are impaired in offenders^11,78^. Offenders may not adjust their behavior based on the available information^79,80^ and avoid losing even small wins because they depend more on the monetary reward. Such negative correlations of socio-economic status and risk-avoidance have been observed in older adults^81^. Moreover, it should be noted that this sample was on probation, potentially influencing their behavior as they may show more socially desirable behavior. However, social desirability was not evident in the self-report questionnaires on aggression (AQ and RPQ) since offenders described themselves as more aggressive as compared to the control group.

The described cognitive deficits may make violent offenders, like other clinical populations^82^, more susceptible to the effects of prefrontal tDCS than non-violent individuals. In the present study, only the offender group exhibited changes in behavior and neural activity after prefrontal tDCS. This heightened susceptibility supports our initial hypothesis and is in line with the growing body of literature on prefrontal deficits in violent offenders^3,83,84^. Prefrontal malfunction in offenders has been related to cognitive deficits^78,85^ and poor decision-making^11^. Facilitating prefrontal activity by tDCS might reduce this deficit. The results of a previous study comparing total earnings in the BART between a cathodal, anodal, and sham group^35^ may support this interpretation. Although the authors find differences in the form of lowered performance in the cathodal group, this is similar to our findings, considering the opposing effects of anodal and cathodal stimulation. Interestingly, in the non-violent control group, decision-making was closer to the reward optimization strategy and hence may reflect a ceiling effect unaffected by tDCS. Thus, tDCS effects may depend on the baseline levels of behavior and neural activation^86^. Moreover, individual differences such as learning capacity or smoking status may influence the direction of modulation effects^87,88^.

### Limitations

The large time gap between the stimulation and the BART entailed the risk of reduced tDCS effects. Studies on tDCS over the motor cortex suggest long-lasting effects 60–120 min after stimulation^89^. It is unclear if the applied stimulation in the current study outlasted the time interval of approximately 40 min until the task started. The current sample appears rather small but it is comparable with other studies in the field^4,79^ and the cross-over design created the optimal sham control group. The offender group also appeared heterogeneous in their offenses, personality traits, psychiatric symptoms, and demographic variables. While representing the actual heterogeneity in this population, it remains unclear which characteristics account for the observed deficits. Larger samples would enable the investigation of offender subgroups. The reported interaction of stimulation and group for adjusted pumps was only marginally significant and the interaction of group and stimulation on dlPFC activity did not hold significance when correcting for multiple comparisons. This may decrease reliability of the findings. Nevertheless, in both cases the post hoc comparisons were significant as predicted by our hypotheses.

## Conclusion

Our results suggest that prefrontal anodal tDCS operates in a baseline-dependent manner with violent offenders being more susceptible. Against the hypothesis, prefrontal tDCS increased risk-taking behavior, but a closer inspection of the data revealed an optimized decision-making performance in offenders. In offenders, activity and connectivity changes in a wide-ranging network of cortical and sub-cortical regions in response to active tDCS support the conclusion that behavioral changes are related to reorganized brain function. Although our results do not indicate that tDCS is suitable for reducing risk-taking in offenders, this proof-of-concept study corroborates the possibility to treat decision-making deficits in this population.

### Supplementary Information

Supplementary Information.

## Data Availability

The datasets generated during and/or analyzed during the current study are not publicly available since making the data publicly available would contradict the agreement with the local ethics committee. Datasets are available from the corresponding author on reasonable request.
