# Supplementary material for: Prefrontal tDCS modulates risk-taking in male violent offenders
Source: Sci Rep. 2024 May 2;14:10087. doi: 10.1038/s41598-024-60795-z (PMC11066090; doi:10.1038/s41598-024-60795-z)
Supplement: Supplementary file 1 — Supplementary Information. [file 41598_2024_60795_MOESM1_ESM.docx]

**Supplementary material**

Prefrontal tDCS modulates risk-taking in male violent offenders (Kuhn et al.)

**Description of the Balloon Analogue Risk Task (BART)**

The applied BART (Fig. 1) is a well-established computerized risk-taking task adapted for use in the MRI (Rao et al., 2008; Wagels et al., 2017). Participants had to inflate virtual balloons of different colors via button press. In experimental trials, a green balloon was inflated. As it grew larger, the monetary reward but also the risk of explosion increased. In case of an explosion, the money from that trial was lost and a new balloon for the next trial appeared. Before each inflation, participants had the option to save their temporal reward to their permanent account. That ended the trial and a new balloon appeared. The time of the explosion varied between trials with a maximum of 12 pumps. On average, the optimal number of pumps would be around 6 pumps to maximize the monetary reward. The explosion probabilities and rewards are equal to those in a previous study (Wagels et al., 2017) but participants were not explicitly informed about the risk-reward structure. During control trials, participants had to inflate a violet balloon until the trial ended itself. The control balloons did not explode and had no influence on the reward. Experimental (n = 33) and control trials (n = 27) were presented in pseudo-randomized order. The BART lasted approximately 30 minutes.


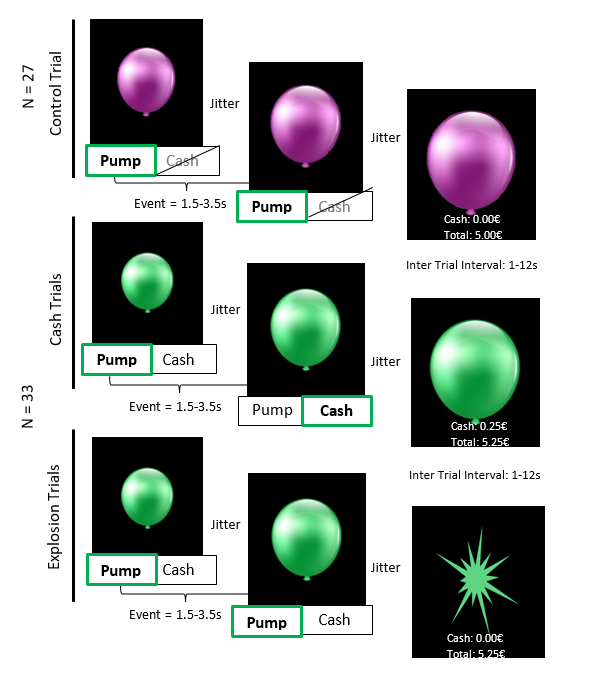


Supplementary Figure 1. Visualization of the different trials in the BART.

**Imaging data acquisition and analysis**

Functional data were collected using a 3T Prisma Scanner (Siemens AG, Erlangen Germany) equipped with a 12-channel matrix head coil. Stimuli were presented on a screen at the back of the scanner that participants saw through a mirror on the coil. They reacted via a MR compatible button box with the right index and middle finger.

During the BART, approximately 830 functional scans were acquired. A spin-echo EPI sequence with the following acquisition parameter were used: TR = 2000 ms; TE = 30 ms, flip angle = 77°, FOV = 192 x 192 mm², matrix size = 64 x 64 mm, 36 slices, slice thickness = 3.1 mm, voxel size = 3 x 3 x 3 mm, interleaved ascending, slice gap 0.8 mm. Anatomical scans were acquired using a T1-weighted MPRGE sequence with the following acquisition parameters: TR = 2300 ms, TE = 2.98 ms, flip angle = 9°, FOV = 256 × 256 mm, 176 slices, voxel size = 1 mm³, interleaved, distance factor: 50%.

Processing and analysis of the fMRI data were conducted with SPM 12 (Wellcome Department of Cognitive Neurology, UK, implemented in Matlab 2017b, Math Works, Natick, MA). For realignment, a two-pass procedure was implemented using the first and a mean EPI scan for reference. Low-frequency drifts were removed using a high-pass filter at 128 seconds. The anatomical scan was co-registered with its mean EPI scan and used for normalization. The functional scans were transferred to standard coordinates of the MNI space. After normalization, all scans were transformed to a voxel size of 2x2x2mm. Finally, scans were smoothed using an isotropic Gaussian Kernel of 8mm full-width-at-half-maximum.

After preprocessing the individual time-series were modelled within the framework of the general linear model (GLM). Different decisions in the BART were modeled as regressors: inflation of the green balloon, cashing out the green balloon and inflation of the violet balloon. Moreover, decisions were parametrically modulated via the explosion risk. Six movement parameters were included as regressors.

**Sample Characteristics**

Supplementary Table 1

*Number of reported psychiatric diagnoses in the violent offender group.*

| Psychiatric Diagnosis | *n* (VO only) |
| --- | --- |
| Depression | 3 |
| Bipolar | 1 |
| ADHD | 2 |
| Addiction | 4 |

Two participants took psychiatric medication (one Olanzapine, one Mirtazapine). Another participant took polamidone for substitution.

**Normality check**

Our models were tested with regard to normality distribution of the residuals. This revealed neither a violation of normality distribution regarding the adjusted pumps model (W = 0.983, p-value = 0.302)) nor in the earning model (W = 0.992, p-value = 0.915).

**Additional results: PANAS**

We compared the mean sum scores of positive and negative affect ratings in the PANAS at the end of the experimental procedure using a repeated measures ANOVA with the between-subjects factor group (controls vs. offenders) and the within-subjects factor condition (active vs. sham). We found no significant main effects or interactions, meaning that the participants were not differentially influenced by the experiment in their affective ratings. (Negative affect: group: *F*(1,33)=1.69, *p*=.20; condition: *F*(1,33)=1.39, *p*=.25; interaction: *F*(1,33)=2.82, *p*=.10); Positive affect: group: *F*(1,32)=.05, *p*=.82; condition: *F*(1,32)=.01, *p*=.92; interaction: *F*(1,32)=.52, *p*=.47). The descriptive statistics can be found in Tables 2 and 3.

| Supplementary Table 2: *Mean (M) Negative Affect ratings and standard error of mean (SEM)* | | | | |
| --- | --- | --- | --- | --- |
|  | Controls | | Offenders | |
|  | active | sham | active | sham |
| M | 11.77 | 12.20 | 14.33 | 11.88 |
| SEM | 0.70 | 0.79 | 1.03 | 0.89 |

| Supplementary Table 3: *Mean (M) Positive Affect ratings and standard error of mean (SEM)* | | | |  |
| --- | --- | --- | --- | --- |
|  | Controls | | Offenders | |
|  | active | sham | active | sham |
| M | 26.46 | 24.00 | 25.00 | 26.88 |
| SEM | 2.35 | 2.68 | 3.79 | 3.00 |

**Model spaces for behavioral analyses**

Supplementary Table 4

*Model space for the adjusted pumps models*

| Model1 <- lmer(aPumps ~ Group* stimulation + session + nExplosion + (1\|ID), data = BART_tdcs) |
| --- |
| Model2 <- lmer(aPumps ~ Group* stimulation + (1\| session)+ nExplosion + (1\|ID), data = BART_tdcs) |
| Model3<- lmer(aPumps ~ Group* stimulation + session*stimulation + nExplosion + (1\|ID), data = BART_tdcs) |
| Model4 <- lmer(aPumps ~ Group* stimulation + session*stimulation + Group:session + nExplosion + (1\|ID), data = BART_tdcs) |
| Model_ Full <- lmer(aPumps ~ Group* stimulation* session + nExplosion + (1\|ID), data = BART_tdcs) |

Supplementary Table 5

*Model space for the earnings models*

| Model1 <- lmer(earnings ~ Group* stimulation + session + (1\|ID), data = BART_tdcs) |
| --- |
| Model2 <- lmer(earnings ~ Group* stimulation + (1\|session) + (1\|ID), data = BART_tdcs) |
| Model3 <- lmer(earnings ~ Group* stimulation + stimulation:session + session + (1\|ID), data = BART_tdcs) |
| Model4 <- lmer(earnings ~ Group* stimulation + stimulation:session + Group:session + session + (1\|ID), data = BART_tdcs) |
| Model _Full <- lmer(Geld_gesamt ~ Group* stimulation * session + (1\|ID), data = BART_tdcs) |

**Correlational analyses**

No significant correlations between the dependent variables (aPumps and total earnings) and the possibly confounding variables (IQ and BDI-II) emerged.

Supplementary Table 6

*Pearson correlation coefficients for both groups*

|  | aPumps | total earnings | verbal IQ | BDI-II |
| --- | --- | --- | --- | --- |
| total earnings | Offenders: .850**  Controls: .869** |  |  |  |
| verbal IQ | Offenders: -.006  Controls: .104 | Offenders: .083  Controls: .009 |  |  |
| BDI-II | Offenders: -.121  Controls: .132 | Offenders: -.102  Controls: .165 | Offenders: .087  Controls: -.412** |  |

Note: ** *p* < .001

Supplementary Table 7

*Pearson correlation coefficients in the whole sample.*

|  | aPumps | total earnings | PPI-R | BIS total | RPQ total | AQ | rdlPFC activity |
| --- | --- | --- | --- | --- | --- | --- | --- |
| total earnings | .872* |  |  |  |  |  |  |
| PPI-R | -.231 | -.163 |  |  |  |  |  |
| BIS-11 total | -.244 | -.232 | .751* |  |  |  |  |
| RPQ total | -.416* | -.359* | .771* | .674* |  |  |  |
| AQ | -.474* | -.327 | .691* | .562* | .846* |  |  |
| rdlPFC activity | .289 | .254 | -.062 | -.111 | -.301 | -.221 |  |

Note: Bonferroni corrected *p* = .05/21 = .002; * *p* < .002

Supplementary Table 8

*Pearson correlation coefficients in the offender group.*

|  | aPumps | total earnings | PPI-R | BIS total | RPQ total | AQ | rdlPFC activity |
| --- | --- | --- | --- | --- | --- | --- | --- |
| total earnings | .850* |  |  |  |  |  |  |
| PPI-R | -.144 | -.088 |  |  |  |  |  |
| BIS-11 total | -.130 | -.177 | .868* |  |  |  |  |
| RPQ total | -.174 | -.130 | .727* | .591* |  |  |  |
| AQ | -.531 | -.311 | .665* | .516 | .580 |  |  |
| rdlPFC activity | .239 | .205 | .146 | .022 | -.213 | .041 |  |

Note: Bonferroni corrected *p* = .05/21 = .002; * *p* < .002

Supplementary Table 9

*Pearson correlation coefficients in the control group.*

|  | aPumps | total earnings | PPI-R | BIS total | RPQ total | AQ | rdlPFC activity |
| --- | --- | --- | --- | --- | --- | --- | --- |
| total earnings | .869* |  |  |  |  |  |  |
| PPI-R | .070 | .208 |  |  |  |  |  |
| BIS-11 total | -.001 | -.001 | .213 |  |  |  |  |
| RPQ total | -.198 | -.120 | .459 | .004 |  |  |  |
| AQ | -.025 | .103 | .274 | -.049 | .710* |  |  |
| rdlPFC activity | .175 | .121 | .044 | .022 | -.142 | -.167 |  |

Note: Bonferroni corrected *p* = .05/21 = .002; * *p* < .002

**Additional fMRI results (main effect of risk)**

Supplementary Table 10

*MNI coordinates of peak voxels in significant clusters of the contrast green > violet.*

| Cluster | Max | Region | *x* | *y* | *z* | *T* | *k* |  |
| --- | --- | --- | --- | --- | --- | --- | --- | --- |
| Cluster 1 | 1 | R MCC | 6 | 26 | 38 | 14.52 | 13970 |  |
|  | 2 | R Insula | 34 | 24 | -4 | 13.20 |  |  |
|  | 3 | R Caudate Nucleus | 12 | 10 | 2 | 12.14 |  |  |
|  | 4 | R Superior Frontal Gyrus | 26 | 50 | 20 | 10.20 |  |  |
|  | 5 | R Middle Frontal Gyrus | 40 | 34 | 34 | 10.14 |  | |
|  | 6 | R Thalamus | 8 | -24 | -4 | 9.74 |  |  |
| Cluster 2 | 1 | R Supramarginal Gyrus | 42 | -42 | 42 | 10.81 | 1920 |  |
|  | 2 | R Angular Gyrus | 30 | -62 | 46 | 6.76 |  |  |
|  | 3 | R Inferior Parietal Lobule | 52 | -34 | 56 | 6.75 |  |  |
|  | 4 | R Precuneus | 14 | -66 | 40 | 5.28 |  |  |
|  | 5 | R Superior Occipital Gyrus | 22 | -60 | 36 | 5.20 |  |  |
| Cluster 3 |  | L Precentral Gyrus | -48 | -6 | 58 | 8.20 | 782 |  |
| Cluster 4 | 1 | Cerebellar Vermis | 4 | -54 | -20 | 7.94 | 713 |  |
|  | 2 | R Cerebellum | 32 | -54 | -28 | 7.43 |  |  |
|  | 3 | L Cerebellum | -6 | -72 | -20 | 6.49 |  |  |
| Cluster 5 | 1 | R Middle Occipital Gyrus | 34 | -84 | 0 | 8.15 | 556 |  |
|  | 2 | R Superior Occipital Gyrus | 22 | -94 | 4 | 6.52 |  |  |
| Cluster 6 | 1 | L Middle Occipital Gyrus | -32 | -90 | -6 | 6.61 | 405 |  |
|  | 2 | L Inferior Occipital Gyrus | -20 | -92 | -4 | 6.58 |  |  |
| Cluster 7 | 1 | L Postcentral Gyrus | -42 | -36 | 52 | 6.87 | 351 |  |
|  | 2 | L Inferior Parietal Lobule | -34 | -48 | 52 | 5.25 |  |  |
| Cluster 8 | 1 | L Middle Frontal Gyrus | -32 | 46 | 18 | 7.24 | 257 |  |
| Cluster 9 |  | L Cerebellum (VI) | -28 | -54 | -26 | 7.06 | 117 |  |
| Cluster 10 |  | Cerebellar Vermis | 4 | -52 | -12 | 7.07 | 93 |  |
| Cluster 11 |  | R Inferior Temporal Gyrus | 50 | -52 | -12 | 5.86 | 68 |  |
| Cluster 12 |  | R MCC | 4 | -24 | 30 | 5.89 | 66 |  |
| Cluster 13 |  | L Precentral Gyrus | -50 | 4 | 22 | 5.69 | 63 |  |

FWE corrected *p* < .05, *k* > 10.


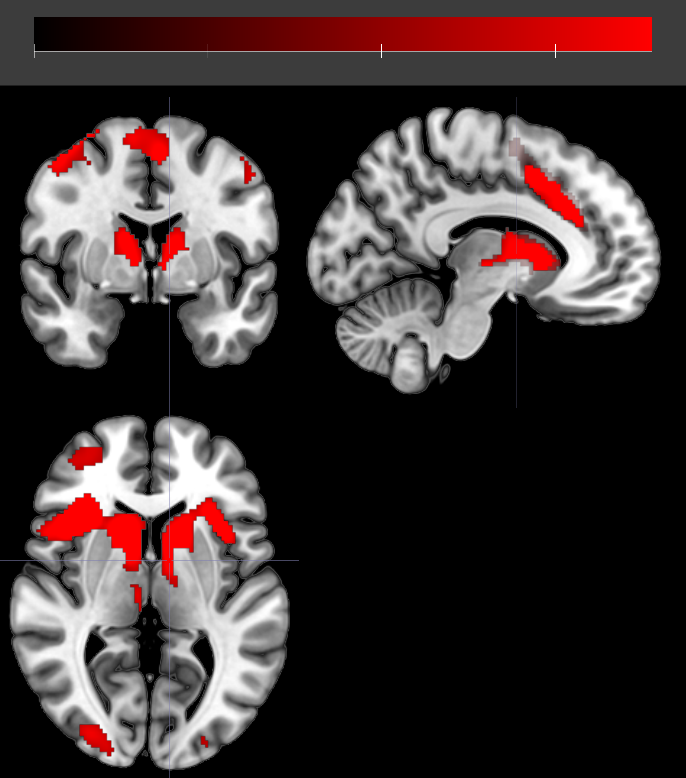


Supplementary Figure 2. Main effect of risk (green > violet balloons) in the BOLD response. FWE corrected p < .05, k > 10.
